# Myths and Methodologies: Understanding the health impact of head down bedrest for the benefit of older adults and astronauts. Study protocol of the Canadian Bedrest Study

**DOI:** 10.1113/EP091473

**Published:** 2024-02-19

**Authors:** Guy Hajj‐Boutros, Vita Sonjak, Andréa Faust, Sharmila Balram, Jean‐Christophe Lagacé, Philippe St‐Martin, Donya Naz Divsalar, Farshid Sadeghian, Teresa Liu‐Ambrose, Andrew P. Blaber, Isabelle J. Dionne, Simon Duchesne, Saija Kontulainen, Olga Theou, José A. Morais

**Affiliations:** ^1^ Research Institute of McGill University Health Centre McGill University Montréal Quebec Canada; ^2^ Faculté des Sciences de l'activité physique, Centre de recherche sur le Vieillissement Université de Sherbrooke Sherbrooke Quebec Canada; ^3^ Department of Biomedical Physiology and Kinesiology Simon Fraser University Greater Vancouver British Columbia Canada; ^4^ Aging, Mobility and Cognitive Neuroscience Laboratory, Department of Physical Therapy, Faculty of Medicine University of British Columbia Vancouver British Columbia Canada; ^5^ Health Research Institute Vancouver British Columbia Canada; ^6^ Centre for Hip Health and Mobility Vancouver Coastal Health Research Institute Vancouver British Columbia Canada; ^7^ Department of Radiology and Nuclear Medicine Université Laval Quebec City Quebec Canada; ^8^ CERVO Brain Research Center Quebec City Quebec Canada; ^9^ College of Kinesiology University of Saskatchewan Saskatoon Saskatchewan Canada; ^10^ Physiotherapy and Geriatric Medicine Dalhousie University Halifax Nova Scotia Canada; ^11^ Division of Geriatric Medicine, McGill University Health Centre McGill University Montréal Quebec Canada

**Keywords:** bedrest, exercise countermeasure, older adults, space health

## Abstract

Weightlessness during spaceflight can harm various bodily systems, including bone density, muscle mass, strength and cognitive functions. Exercise appears to somewhat counteract these effects. A terrestrial model for this is head‐down bedrest (HDBR), simulating gravity loss. This mirrors challenges faced by older adults in extended bedrest and space environments. The first Canadian study, backed by the Canadian Space Agency, Canadian Institutes of Health Research, and Canadian Frailty Network, aims to explore these issues. The study seeks to: (1) scrutinize the impact of 14‐day HDBR on physiological, psychological and neurocognitive systems, and (2) assess the benefits of exercise during HDBR. Eight teams developed distinct protocols, harmonized in three videoconferences, at the McGill University Health Center. Over 26 days, 23 participants aged 55–65 underwent baseline measurements, 14 days of −6° HDBR, and 7 days of recovery. Half did prescribed exercise thrice daily combining resistance and endurance exercise for a total duration of 1 h. Assessments included demographics, cardiorespiratory fitness, bone health, body composition, quality of life, mental health, cognition, muscle health and biomarkers. This study has yielded some published outcomes, with more forthcoming. Findings will enrich our comprehension of HDBR effects, guiding future strategies for astronaut well‐being and aiding bedrest‐bound older adults. By outlining evidence‐based interventions, this research supports both space travellers and those enduring prolonged bedrest.

## 1 INTRODUCTION

High levels of human performance are essential for successful spaceflight missions. However, weightlessness due to spaceflight can negatively affect multiple physiological systems leading to loss in bone density, muscle mass, strength, cardiorespiratory fitness, sensorimotor and possibly cognitive functions (Hedge et al., 2022). These changes can severely affect the ability of astronauts to perform mission‐related tasks and increase the risk of injury. Thus, it is essential to develop effective countermeasures to reduce the negative consequences of spaceflight and to maintain crew health and fitness.

Six‐degree head down tilt bedrest (HDBR) has long been used as an analogue to microgravity permitting a more detailed investigation of physiological mechanisms as well as the development and testing of various ground countermeasures (e.g., exercise, nutrition, etc.) before re‐testing them in a microgravity environment (Scott et al., 2021). Although exercise is the main countermeasure in manned space missions, namely to sustain aerobic capacity, musculoskeletal structure and cardiovascular function, so far there is no single exercise that has proven to be entirely effective in maintaining or restoring cardiovascular and musculoskeletal functions to preflight levels after prolonged spaceflight (Hedge et al., 2022). However, it should be noted that HDBR studies also have great relevance and applications in the terrestrial clinical context. The dramatic changes that the human body undergoes during bedrest due to illnesses are similar to those seen over decades of normal ageing (Kehler et al., 2019). Bedrest in otherwise healthy older individuals can lead to several deleterious physiological effects including loss of muscle mass and strength (English & Paddon‐Jones, 2010), impaired postural control and bone structure (Mulder et al., 2014), reduced orthostatic tolerance, and even altered cognitive function (Balbim et al., 2024).

With space agencies planning to conduct longer missions and more complex explorations, understanding the biological changes that occur with exposure to microgravity is of the utmost importance. Further, it is imperative to discern how bedrest gives rise to health problems as a model of accelerated ageing, while measuring the impact of exercise as a countermeasure. This first Canadian head down bedrest study started with the operating grant competition: ‘Understanding the Health Impact of Inactivity’ launched jointly by the Canadian Institute of Health Research (CIHR), the Canadian Space Agency (CSA) and the Canadian Frailty Network in 2017. Subsequently, another national competition was launched by CSA and CIHR to host the participants and execute a study meeting the objectives of all the eight successful teams of the competition as well as the standard measurements required by CSA. The eight research teams were from several universities across Canada including McGill, University of Waterloo, Sherbrooke University, Laval University, Dalhousie University, University of Saskatchewan, University of British Colombia and Simon Frazer University. After obtaining the contract with CSA, the team of Montreal from the McGill University Health Center, organized three half‐day videoconferences with all parties to define the integrated study in which the host site would collect data from participants enrolled in the protocol. This integrated study will lay the foundation for a detailed description of conducting HDBR, inflight countermeasures and recovery programmes, as well as new protocols for pre‐ and post‐HDBR/spaceflight health monitoring. This study stands out because it is the first time a bedrest study has incorporated this type and duration of exercise protocol. It is also one of the first studies in an older age group. Unlike most bedrest studies that primarily involve young adults, this investigation would provide additional information by targeting an older demographic.

## 2 OBJECTIVES OF THE EIGHT RESEARCH TEAMS

Objective 1: Our first objective was to determine the alterations in body composition, physical capabilities and postural stability linked to HDBR both with and without exercise interventions. Furthermore, we aimed to examine how HDBR influences cognitive functions across various domains like memory, attention and executive function, alongside sleep quality, and to discern the impact of exercise interventions on these factors.

Objective 2: The second objective was to analyse shifts in diverse blood parameters, encompassing haematology, hormonal levels, neurotrophic factors, inflammatory markers, bone metabolism, muscle protein dynamics and overall nutritional health in response to HDBR and exercise interventions. We sought to quantify changes in biological age via DNA methylation assessments. Additionally, we intended to monitor alterations in protein profiles, bone turnover markers across blood, saliva and urine samples. Our aim further included collecting faeces samples to investigate microbiome changes and saliva samples to determine shifts in hormonal concentrations and protein synthesis rates.

Objective 3: Our third objective was to evaluate modifications in cellular processes related to muscle protein synthesis, breakdown, muscle volume and strength, including inflammation due to both HDBR and exercise interventions. The objective also involved assessing changes in cardiac functionality, alterations in muscle and bone structures, infiltration of fat in muscles and bones, as well as neuromuscular activities in the lower limbs and muscle‐pump baroreflex responses induced by HDBR and exercise interventions. Finally, we intended to examine variations in brain structure and functionality influenced by both HDBR and exercise interventions.

Herein, we wish to describe in more detail the overall structure of the integrated study including the CSA‐required standard measures (blood biomarkers, aerobic capacity, muscle strength, body composition and bone density). The list of standard measures was provided under the contract and originated from the *Guidelines for Standardization of Bedrest Studies in the Spaceflight Context*: https://iaaspace.org/product/guidelines-for-standardization-of-bed-rest-studies-in-the-spaceflight-context/. These results as well as those of the specific measurements collected for each of the successful teams (Table 1) will be published in separate publications.

**TABLE 1:** Schedule of procedures of each visit.

		Baseline						Head down bedrest														Recovery							4 w	4 m
Testing	S	0	1	2	3	4	5	1	2	3	4	5	6	7	8	9	10	11	12	13	14	1	2	3	4	5	6	7	1	1
Physical exam	X																													
Physical activity monitoring	X											X	X	X	X	X	X	X				X	X	X	X	X	X	X	X	
Hexoskin			X	X				X	X				X	X					X	X		X	X	X	X		X		X	X
Radar in‐bed body monitoring								X	X				X	X					X	X										
Cognitive performance			X				X							X								X						X		X
Physical function and sensorimotor testing			X	X																		X					X		X	X
Tilt test							X															X					X		X	X
Postural equilibrium control + Vestibular assessment standing			X																			X							X	
Vertical jump + Biodex + EMG							X																X			X			X	X
DXA	X				X																			X					X	X
Stand test				X																		X							X	
Supine‐to‐stand test				X			X															X								
Baroreflex test									X	X								X												
Organization of sensory motor control test			X																			X							X	
Heavy water					X	X	X	X	X	X	X	X	X	X	X	X	X	X	X	X	X	X	X	X	X	X	X			
Tracer infusion								X		X											X									
REE			X						X					X							X									
${\dot{V}}_{O_{2}\mathrm{peak}}$	X			X																			X				X		X	X
Cardiac/vascular ultrasound				X														X					X						X	
Sleep quality			X	X			X	X						X	X						X	X				X	X			
Body and heart MRI			X																	X										
Brain MRI						X																	X							X
pQCT					X																			X						
HR‐pQCT				X																			X						X	
Blood volume					X														X											
Blood/urine	X		X		X	X		X		X			X	X	X	X			X	X	X	X		X	X		X	X	X	X
OGTT						X					X					X				X							X			
Saliva collection			X	X	X		X	X	X	X	X	X	X	X	X	X	X	X	X	X	X	X	X	X	X	X	X	X	X	X
Breath analyser	X																													
Faeces collection			X					X	X	X	X	X	X	X	X	X	X	X	X	X	X			X			X		X	X
Muscle biopsy					X			X		X					X						X							X	X	

S, screening; 4 w, 4 weeks; 4 m, 4 months. DXA, dual energy X‐ray absorptiometry; HR‐pQCT, high‐resolution pQCT; MRI, magnetic resonance imaging; OGTT, oral glucose tolerance test; pQCT, peripheral quantitative computed tomography; REE, resting energy expenditure.

## 3 METHODS

The study protocol follows the SPIRIT guidelines (Chan et al., 2013).

### 3.1 Ethics approval

The study received ethical approval by the Research Ethics Board of the MUHC, under study no. 2021–7170. A total of ten $10,100 was given to the participants following the completion of the study.

### 3.2 Clinical trial registration

This study was registered at ClinicalTrials.gov and assigned the identification code NCT04964999. All specimen collections and measurements were performed and supervised by experienced staff: qualified kinesiologists, psychologists, healthcare professionals, physicians, and so forth, at the Research Institute of McGill University Health Centre (RI‐MUHC)‐Centre for Innovative Medicine (CIM), Lindsay Gingras Rehabilitation Centre and the Douglas Mental Health University Institute in Montreal, Canada.

### 3.3 Overview of the study design

This study was a two‐arm randomized controlled trial where participants and assessors were not blinded. The recruitment started mid‐April 2021 and the first participant admitted to the study was on the 12 July 2021. A total of 23 participants (11 females and 12 males) were recruited from the greater Montreal area through digital and print advertising. Once a candidate shared their interest in participating (calling the devoted research phone or by sending an email of interest to the study address), they first underwent a telephone screening that included reviewing the study protocol, the inclusion and exclusion criteria, psychological questions about their emotional state as well as their nutrition and physical activity. If the candidate passed the initial phone screening, they were invited to the second, in‐person screening visit at the RI‐MUHC‐CIM. During this visit, the study requirements were re‐explained in detail and written informed consent was obtained before any medical examination by our research coordinator, blood and urine collection, physical function measurement, and psychological assessments were performed. Only the participants that met the inclusion criteria were invited to participate in the study. During the screening visit, participants received an accelerometer to measure their physical activity for 7 consecutive days. Participants eligible to participate in the study were randomized into the exercise group or the control group using a computer‐generated random number. Following the recruitment and randomization process, participants were admitted to the CIM for 26 days. Participants were admitted in cohorts of five to six individuals, with admissions staggered every 2 days. Once a group completed their 26‐day inpatient period, another group of five to six participants was admitted, maintaining the staggered approach to ensure no overlap occurred. We successfully completed a total of four cohorts, with the entire duration to finish the 26‐day inpatient stay spanning approximately 6 months. Upon the participants’ arrival, the evening prior to the start of the inpatient stay was referred to as Day 0. Participants were free to circulate for the initial 5 days of baseline data collection (BDC), during which they underwent baseline measurements (Table 1; BDC). Thereafter, they began the 14 days of HDBR while being on a weight‐maintaining diet (see section 3.6). Following the 14 days of bedrest, participants repeated all baseline measurements while recovering during 7 consecutive days. Results were entered into a data collection software, REDCap (Research Electronic Data Capture), which is a secure web application for building and managing online surveys and databases. The sole location hosting both the study and participants was the McGill University Health Center.

#### 3.3.1 Participant inclusion and exclusion criteria

Inclusion criteria comprised being 55–65 years old, being postmenopausal for female participants (no menses for at least 1 year, or documented ovariectomy, and a serum follicle‐stimulating hormone above 30 IU/l), having a height between 158 and 195 cm with a body mass index between 20 and 30 kg/m^2^, being physically and mentally healthy, and successfully passing the psychological (Positive and Negative Affect Schedule (PANAS) and General Health Questionnaire (GHQ) questionnaires) and medical screening. Exclusion criteria included electrocardiogram abnormalities, testing HIV positive, testing hepatitis B or C positive, having anaemia (haemoglobin or ferritin values outside range for sex), a family history of thrombosis or bone mineral density measured by dual energy X‐ray absorptiometry (DXA) scan greater than 2.0 standard deviations (≥2 *T*‐score), taking medications that affect blood pressure or muscle mass, having claustrophobia, being sedentary (more than 7 h of sitting), making special dietary requests (e.g., vegetarian, vegan or other special diets), having devices implanted under the skin (pacemakers and intra‐cardiac devices, infusion pumps, cerebral artery aneurysm clips, dental implants, a tissue expander, etc.), having given blood in the past 3 months before the onset of the study, smoking (tobacco and/or marijuana mixed with tobacco (tetrahydrocannabinol) within 6 months prior to the start of the study, having abused drugs, medicine or alcohol within up to 30 days prior to the start of the study (alcohol: >10 drinks a week, with more than two drinks a day most days; drugs: tetrahydrocannabinol, cocaine, opiates, amphetamines/methamphetamines, benzodiazepines, barbiturates, buprenorphine, creatine, methadone metabolite, oxycodone, phencyclidine), participation in another study within 2 months before study onset, and testing COVID‐19 positive 1 week to 24 h before study start date. The MUHC research team was in charge of allocation and followed the CSA recommendation for the intervention. None of the team members were blinded.

#### 3.3.2 Course of the HDBR, recovery and follow‐up periods

Participants, regardless of their group allocation and in accordance with the guidelines for bedrest studies, were able to freely move in bed and stretch on their own as well. Aside from the exercise sessions, there was no difference between the two groups. Participants of the exercise group took part in a daily structured exercise programme. However, the control group received daily physiotherapy exercises such as stretching, motion therapy and massages provided by the study physical therapist when necessary (if any low back pain or discomfort was mentioned). Every participant received regular visits by the nurse, physician, dietician and research staff. Following the HDBR, participants underwent 7 days of recovery (before being discharged home), during which they were permitted to ambulate under supervision. They were involved in a structured rehabilitation, and baseline testing was repeated (Table 1). Post recovery period, participants were scheduled for 4‐week and 4‐month follow‐up visits to undergo various repeated measurements (Table 1). They arrived once again to the facility the evening prior and spent the night at the CIM. Throughout the study, we monitored participants for the presence of any adverse events by asking them how they felt and if they experienced any health symptoms such as body pains, joint swelling, muscle spasms, headache, loss of appetite, dizziness, confusion and so forth. In case of a clinically relevant incident (including, but not limited to changes from baseline) after enrollment, the principal investigator and/or study physician would determine if any changes in the participant's management were needed. The latter also included ending the study, in the event that they experienced serious conditions. Every new clinically relevant finding was reported as an adverse event to the Research Ethics Board.

#### 3.3.3 Facility set‐up

For the inpatient stay, participants of the same sex were accommodated in a double‐bedded room separated by a curtain for privacy during specific testing. However, participants were encouraged to interact with each other to discuss and share their experiences. During bedtime, participants were moved into single bedrooms where they stayed the entire night in order to have privacy. One of the personnel was continuously available on site, and each participant had a device to call in case of need or an emergency. Bed sensors were installed under each participant's mattress to assure that they are always maintaining their HDBR position. Once a participant raised their head/torso, an alarm inside the participant's room and with the study coordinator's monitor would start. These monitors were used 24 h during the 14 days of bedrest. Participants were allowed to have visitors at the end of the day when all scheduled measurements/tests were completed. The study staff would take participants occasionally outside of the facility during the HDBR period using a stretcher, for them to enjoy some fresh air. During the HDBR, participants were taken on a stretcher to a private washroom for bowel movement and urination. They were trained on how to use urinals and bedpans in HDBR position during the baseline period.

### 3.4 Exercise training intervention

**FIGURE 1:**
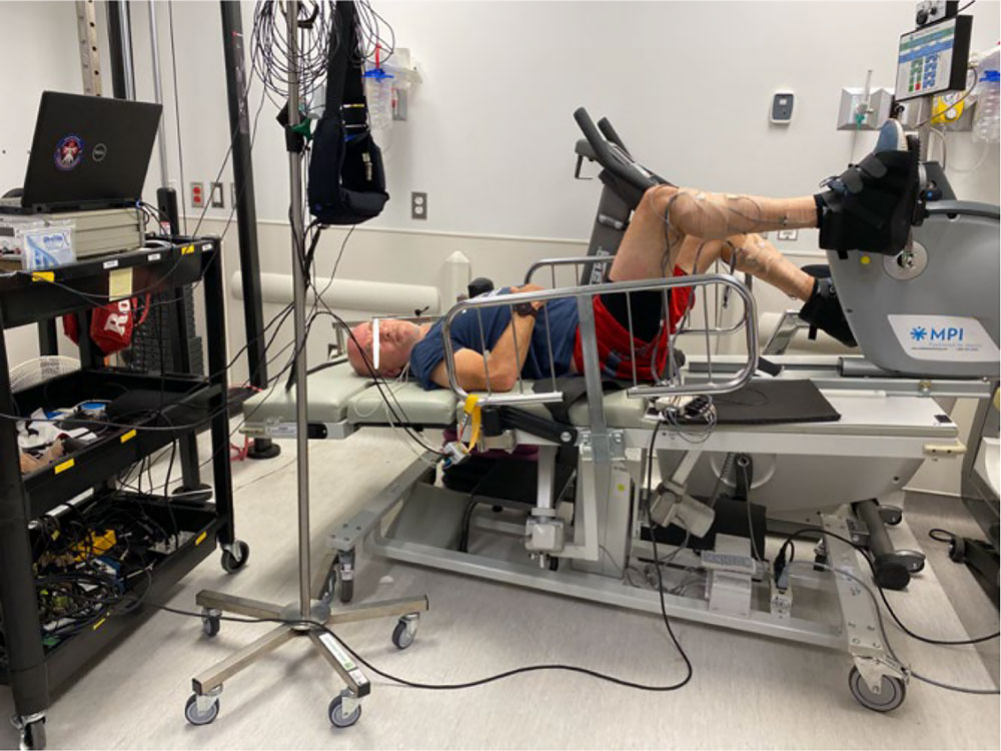
Aerobic and HIIT exercise.

**FIGURE 2:**
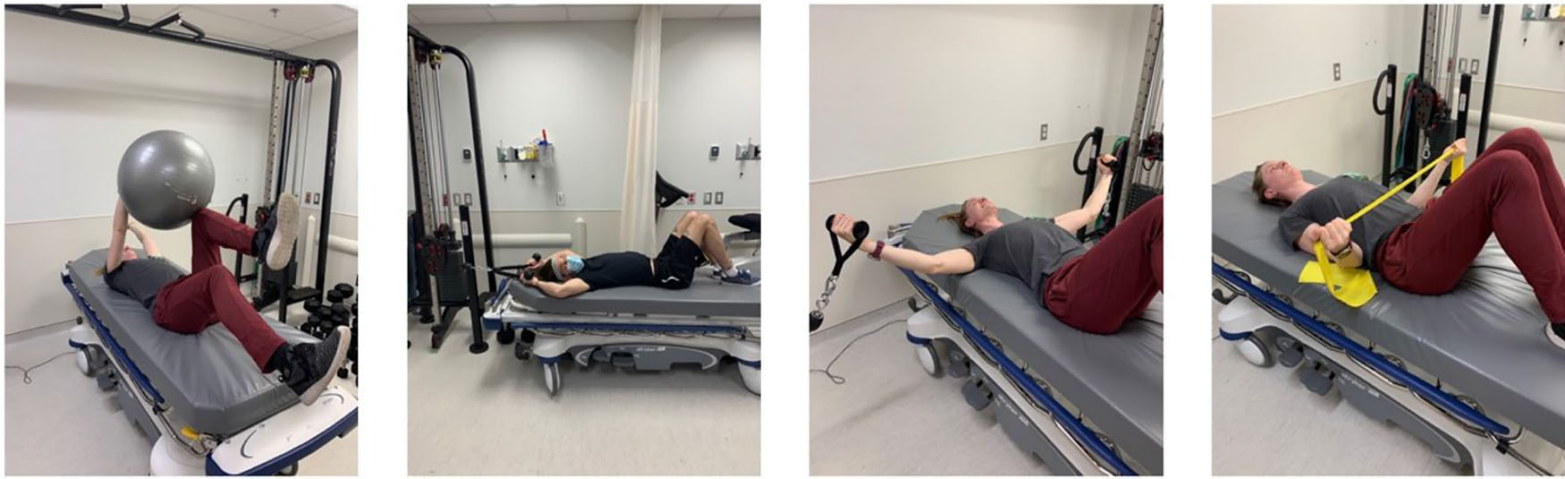
Upper body resistance exercises.

**FIGURE 3:**
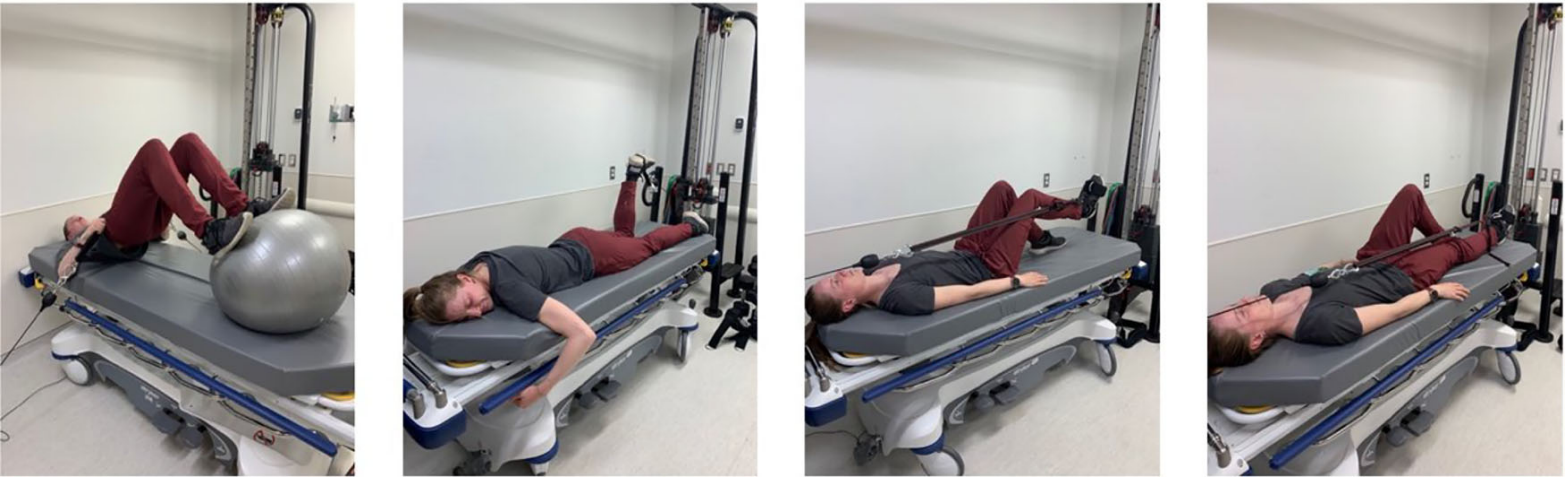
Lower body resistance exercises.

The standardized countermeasure procedure used in the present study was pre‐established by the CSA and adjusted by the MUHC kinesiologist in collaboration with the CSA colleague. The countermeasure comprised three sessions per day of either high‐intensity interval training (HIIT) or low‐intensity cycling as well as strength exercises for the lower and upper body. This resulted in a total of 60–75 min of physical exercise per day. All exercises were completed in a head‐down tilt or horizontal position with available equipment as per a planned schedule (Figure 1). The intensity of the exercise countermeasures was individually adjusted according to participant performance and tolerance. No maximal repetition test was conducted before the intervention. Instead, the session involved performing three sets of 12–15 repetitions until failure, as determined by the overseeing kinesiologist. Additionally, each participant's file documented the rate of perceived exhaustion, heart rate and the overall enjoyability of the session. Upper body exercises comprised external rotation, lateral pull‐down, chest fly and the dead bug (Figure 2). Lower body exercises included hip raises, unilateral leg presses, ankle pumps and leg curls (Figure 3). At different points during the exercise programme, heart rate was monitored in addition to the perception of the intensity using the Borg scale. Additionally, muscle activity in the lower legs was measured using surface electromyography (EMG), where electrodes were placed on the skin overlying lower limb muscles to detect the electrical activity. Moreover, the energy expenditure of three different HIIT sessions throughout the 14‐day HDBR were measured using a portable indirect calorimetry system (MetaMax 3B‐R2®, CORTEX Biophysik, Leipzig, Germany). The countermeasure programme was designed to address several key physiological systems affected by HDBR or spaceflight. First, in order to break up sedentary time, three 60–75 min exercise sessions, with a 4‐h interval between sessions, were performed each day. This has been shown to be beneficial for cardiometabolic health (Bergouignan et al., 2016; Healy et al., 2008). Second, HIIT is known to maintain or improve aerobic fitness as well as reduce cardiometabolic disturbances in clinical populations (e.g., type 2 diabetes) (Cassidy et al., 2016). Additionally, implementing HIIT may improve the baroreflex response, which becomes altered during HDBR (Convertino, 1997). Overall, the cycling exercises as well as upper‐ and lower‐body resistance protocols were utilized to resemble high‐intensity, low‐volume programmes which have been shown to mitigate the negative effects of HDBR and spaceflight on musculoskeletal and cardiovascular outcomes in a younger population (English et al., 2020; Ploutz‐Snyder et al., 2018). The detailed version of the protocol is described in a previous review published by Hedge et al. (2022), and a specific description of each session is shown in Table 2.

**TABLE 2:** Proposed exercise countermeasure protocol by the Canadian space agency.

Week 1	Day 1	Day 2	Day 3	Day4	Day 5	Day 6	Day 7
Session 1	Resistance, upper	Cont. aerobic (30)	Progressive aerobic	Cont. aerobic (30)	HIIT	Cont. aerobic (30)	HIIT
Session 2	Progressive aerobic	Resistance, lower	Cont. aerobic (15)	Resistance, upper	Progressive aerobic	Resistance, upper	Cont. aerobic (15)
Session 3	HITT	Progressive aerobic	HITT	Progressive aerobic	Resistance, lower	Progressive aerobic	Progressive aerobic

Week 2	Day 8	Day 9	Day 10	Day 11	Day 12	Day 13	Day 14
Session 1	Cont. aerobic (15)	HIIT	Cont. aerobic (30)	HIIT	Cont. aerobic (30)	HIIT	Cont. aerobic (15)
Session 2	Resistance, upper	Cont. aerobic (15)	Resistance, lower	Cont. aerobic (15)	Resistance, lower	Progressive aerobic	Cont. aerobic (30)
Session 3	Progressive aerobic	Progressive aerobic	Progressive aerobic	Progressive aerobic	Progressive aerobic	Resistance, upper	Progressive aerobic

### 3.5 Recovery period

**FIGURE 4:**
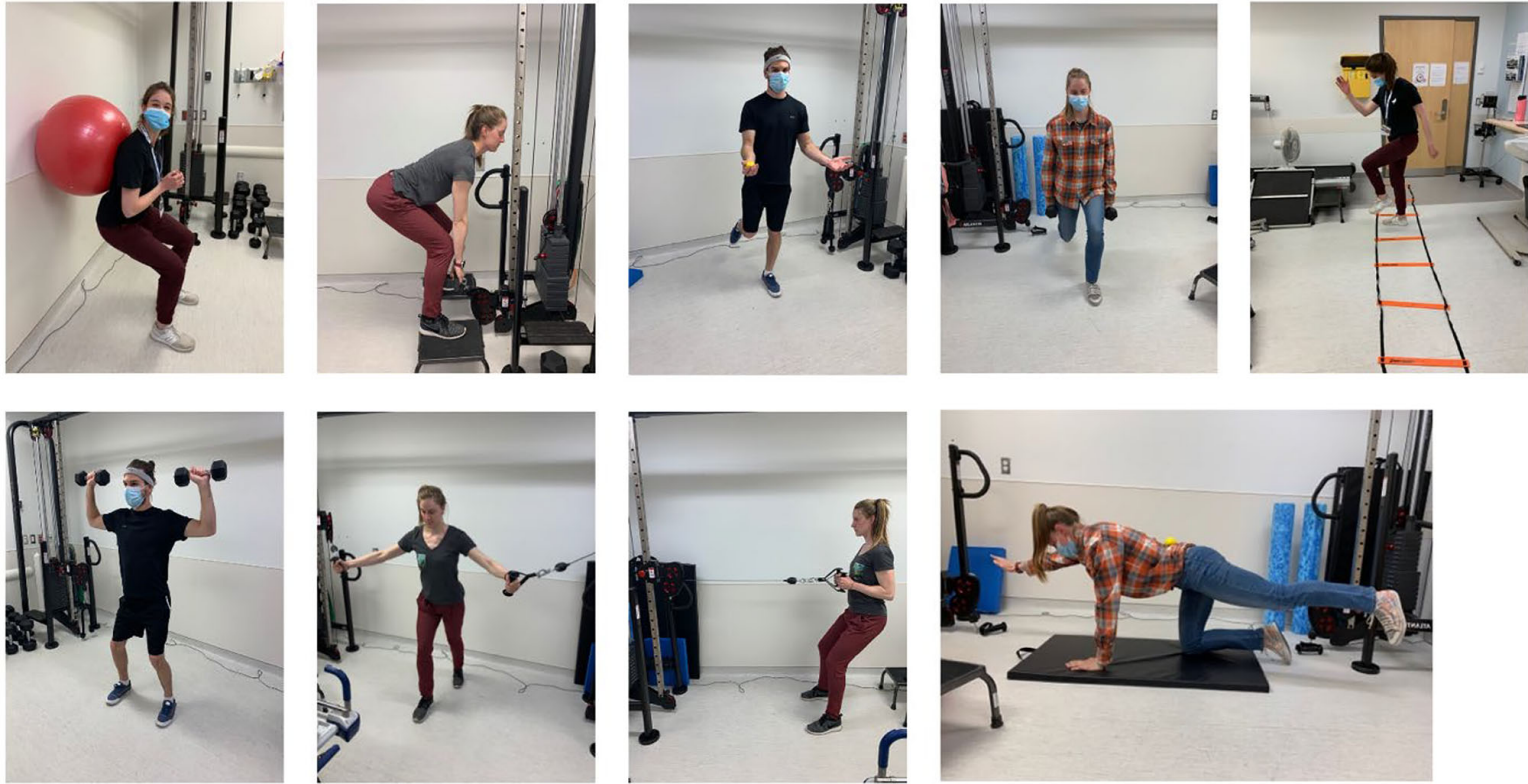
Recovery exercises.

During the 7‐day recovery period, participants of both exercise and control groups underwent supervised rehabilitation exercises in addition to normal ambulation and typical daily activities. Two 15‐min aerobic exercise sessions were completed each day on a treadmill or on an ergocycle in the event that the participant was unable to walk upright. The exercise session consisted of a 3‐min warm‐up at 40% heart rate reserve (HRR), 9 min of exercise at 60–80% HRR, and 3‐min cool‐down at 40% HRR. In addition to the aerobic sessions, participants were asked to perform strength and stability exercises for either the upper or lower body on alternating days. The lower‐body exercise session started with a warm‐up using an agility circuit followed by squat‐lunges, dead‐lifts, jumping on to a step and ending with balancing on one leg. The upper‐body session also began with an agility circuit warm‐up using rope ladders, followed by cable rowing, chest and military press as well as lateral pull down. Front/side planks and quadruped exercises were also used for core stability on a daily basis. Two repetitions of the agility circuit were used for all warm‐ups, and three repetitions of up to 30 s were performed standing on each leg as well as in a plank position. Three sets of 10–12 repetitions were used for the other strength exercises. These exercises have been previously performed by the CSA exercise specialist with astronauts following their mission. Depending on their ability to perform the exercises, participants were asked to provide their rate of perceived exhaustion after each exercise in addition to heart rate. On days 1 and 2, some participants experienced drops in blood pressure during the day and adaptation of the exercise protocol was necessary to avoid any fall. Some exercises were performed seated (on ergo cycle or chair). Figure 4 illustrate exercises during the recovery session.

### 3.6 Study diet and dietary supplementation

Participants received an adjusted diet throughout the study depending on their needs. As their resting metabolic rate (RMR) was measured by indirect calorimetry during the baseline period, their energy intake matched RMR × 1.1 to which a surplus of 10% was added to compensate for the thermic effect of food as proposed by ESA/NASA, which should be close to measured RMR ×1.2. During the baseline and recovery phases, their energy intake was set at RMR × 1.5, including the thermic effect of food. Energy intake of participants in the exercise group was corrected by the energy expenditure of exercise that was measured by an indirect portable calorimeter during their preparation session of the baseline phase, meaning that those participants needed to consume more calories per day. Participants’ appetite and weight were taken into consideration and food intake was adjusted to maintain their weight as much as possible throughout the study. The daily menu was composed of high‐quality protein of value and intake appropriate for this age‐group of 1.2 g/kg/day with at least 0.4 g/kg/meal with a balanced macronutrient content with vitamins and minerals. They received a multivitamin and mineral preparation to meet a pre‐established calcium intake of 1200 mg and vitamin D of 1000 IU per day. Since energy expenditure decreased due to bedrest, some ended up with fairly low energy intakes (≤1500, women especially); they needed some protein‐enriched foods and/or supplements to reach the protein intake goal. Moreover, a significant challenge encountered during the 14‐day bedrest period was a noticeable loss of appetite among participants. Many individuals struggled to consume the entirety of the food provided, necessitating adjustments by dieticians, even with the supplementation offered. All food ingested during the 26‐day inpatient stay was monitored using the software Keenoa (a phone application that recognizes food items from a picture), to control for caloric and macronutrient intake. During this period, participants were asked to fill out a food intake and satiety questionnaire. Additionally, at the beginning of the study and follow‐up visits, participants were also be asked to fill out a food frequency questionnaire and to track their nutrient intake for 3 days with the Keenoa application (Bouzo et al., 2021; Moyen et al., 2022). In case any of the participants did not have a phone with a camera, they were provided with a hard copy of the 3‐day food diary. On one day in baseline and one day in HDBR, blood samples were obtained 1 h prior to and 1 h after completing the meal to measure appetite‐related hormones, along with a pre–post meal survey.

Participants had the opportunity to choose between two or three meals the night prior depending on their preference for breakfast, lunch and dinner. They also had a large variety of snacks from which they could choose in case they did not meet the daily dietary recommendations. Participants had the option to decide whether they preferred to eat lateral recumbent or prone. A table was placed at the top of the bed and the participants were permitted to slide to the edge of the bed keeping their head down. Bowel movements and fluids were monitored daily to avoid any risk of dehydration and assure that participants were not constipated. Recommended values of nutrient intake levels are defined in Table 3.

**TABLE 3:** Recommended values of nutrient intake.

Nutrient, energy (%TEE) and macronutrients	Adequate intake
Total fat	30–35%
Saturated fatty acids	≤10%
Monounsaturated fatty acids	≥10%
Polyunsaturated fatty acids	≥7%
Protein	1.2 g/kg BW/day
Carbohydrates	50–60%
Total fibre	≥30 g/day

Abbreviations: TEE, total energy expenditure; BW, body weight.

## 4 EXPERIMENTAL MEASURES

### 4.1 Physical examination and anthropometry

Before the start of any physical activity measurements, one of the study physicians performed a physical examination with vital signs, a complete electrocardiogram (ECG) and blood oxygen saturation, to ensure that there were no medical contraindications to any of the tests performed during the study. Additionally, a standing blood pressure measurement was performed to confirm the absence of orthostatic hypotension. Participants’ weight, height and several body segment circumferences were collected.

### 4.2 Physical activity monitoring

Data on physical activity patterns during a typical week were collected using an Actigraph accelerometer (ActiGraph, Pensacola, FL, USA). This monitoring was part of the screening to ensure the appropriate level of physical activity. The accelerometer data were additionally used to confirm the results obtained by the Get Active questionnaire. The Actigraph was worn on the wrist during daytime and night‐time for 6 days. This provided us with the type and amount of physical activity that the participant is performing.

### 4.3 Twenty‐four‐hour activity monitoring

The wearable sensor shirt (Hexoskin, Health Sensors & AI, Montreal, QC, CA) was worn by participants for 24‐h periods in baseline, HDBR and recovery. The device recorded continuous physiological signals including an electrocardiogram, respiratory plethysmography and tri‐axial acceleration.

### 4.4 Radar in‐bed body monitoring

An RF/mm‐wave radar device was set up beside the participants’ bed during the 14‐day bedrest period. The radar was positioned to monitor the participants while they were in bed to extract movements, heart rate, and respiration.

### 4.5 Cognitive performance

Cognitive performance was measured by using the NIH Toolbox Cognition Battery, a comprehensive neuropsychological battery composed of the Flanker Inhibitory Control and Attention Test to assess executive function and attention as well as emotional measures (https://www.healthmeasures.net/explore‐measurement‐systems/nih‐toolbox/intro‐to‐nih‐toolbox/cognition); Picture Sequence Memory Test and the Auditory Verbal Learning Test to assess episodic memory; Picture Vocabulary Test and Oral Reading Recognition Test to assess vocabulary knowledge and oral reading skills; Pattern Comparison Processing Speed Test to assess information processing speed and List Sorting Working Memory Test to assess the working memory. All the measurement were performed lying down with a 6° Head down tilt (HDT) even during baseline and recovery to maintain consistency throughout the study.

### 4.6 Functional tests

Lower extremity physical performance was assessed by the Short Physical Battery Test (Pavasini et al., 2016). Functional capacity was assessed using the 40 m gait speed test (Wright et al., 2011), timed‐up‐and‐go test (Beauchet et al., 2011), four‐square step test (Moore & Barker, 2017) and uni‐pedal stance test (Hurvitz et al., 2000) were used to assess dynamic and static balance, respectively. A Berg balance was used to assess static balance and fall risk in adults (Lima et al., 2018). During these tests, subjects could stop and rest if necessary, and they were closely monitored to ensure safety. Participants were additionally asked to fill out the Frailty Index questionnaire (Searle et al., 2008). The above tests were selected for their validity, prognostic value and broad use in the ageing literature.

### 4.7 Muscle strength testing

Three different techniques were employed to assess muscle strength. A quantitative multi‐joint muscle Biodex dynamometer (Isokenetics Systems, Shirley, NY, USA) was used to assess muscular strength of the thigh (quadriceps and hamstrings) muscles as well as muscles of the ankle. Isokinetic and isometric muscle strength was used in both ankle and knee flexion and extension. The participants were asked to perform maximal voluntary isometric contractions and isokinetic contractions to assess strength and power. Knee flexion and extension torques were assessed under isokinetic conditions, employing standard constant angular velocities of 60°/s and 180°/s. The test comprised a sequence of five extension and flexion movements at 60°/s and 10 attempts at 180°/s, with three preliminary trials involving moderate muscle engagement. Only movements where participants achieved peak muscle torques were considered in the analysis. To enhance measurement reliability, we ensured that the selected peak torque for analysis did not significantly differ from other values. This verification was conducted promptly after each measurement session using Biodex software. Participants started with the ankle joint and then moved to the knee. Additionally, a hand‐held (dynamometer Jamar™, JLW Instruments, Chicago, IL, USA) was used to assess handgrip strength. Standardized verbal encouragements were provided throughout the tests. Subjects were familiarized with the equipment and proper exercise technique prior to the assessment.

### 4.8 Tilt test

The tilt test was used to assess orthostatic tolerance before and after bedrest. Subjects were instrumented while in a supine position, which was maintained while baseline data were collected for 10 min. The table (HUT™ Tilt Table, Medical Positioning Inc., Kansas City, KS, USA) was then tilted to 80° placing subjects in a head‐up tilt position at a rate of approximately 7°/s. Subjects remained in this position for 15 min or until they exhibited symptoms of presyncope (Kocyigit et al., 2019). In order to maintain the HDT position during post‐evaluation, participants were transported using hospital stretchers with a 6° HDT until the test started.

### 4.9 Postural equilibrium control

Postural stability was evaluated using a computerized dynamic posturography system. During this test participants’ blood pressure was constantly monitored. The test was performed on day 1 of recovery. However, due to the difficulties standing (vestibular and blood pressure drops), we had difficulties with five to six participants to measure their actual balance.

### 4.10 Vestibular assessment during a standing task

Galvanic vestibular stimulation was used to assess the control of standing balance by evoking vestibulospinal responses. When applied in a bipolar and binaural manner (electrodes on the mastoid processes behind both ears), galvanic vestibular stimulation evoked a brief depolarization of vestibular afferents on one side, and hyperpolarization on the other side. Measurements were performed at baseline and on day 1 of the study following the postural equilibrium control. We experienced the same difficulties previously described with drop of blood pressure on day 1 making the results difficult to interpret for a few participants.

### 4.11 Vertical jump

Vertical jump was used to assess whole‐body power output (Aragón, 2000). Prior to the vertical jump, the participants performed a warm‐up session and were instructed to perform three warm‐up squats. A participant was then instructed to perform two to three practice countermovement jumps at 50% of maximum effort to ensure proper technique (hands on the side) and warm‐up. Once the participant warms up and proper technique is demonstrated, the operator allows the participant to attempt three maximum effort jumps. The subject rested 60–90 s in between each jump or longer if the subject desired more time between tests.

### 4.12 Dual energy X‐ray absorptiometry scan

This non‐invasive technique was used to measure body composition and provided a quantification of the major body compartments including bone mineral and soft tissue, with the latter divided into fat and fat free tissue (Clasey et al., 1997). It involved lying on an open scanner (Lunar Progidy DXA, GE Healthcare, Madison, WI, USA) for approximately 30 min while two X‐ray beams with different energy levels aimed at the subject's bones, fat mass and muscle. This method presents a low within‐subject coefficient of variation (approximately 1.5%) and strongly correlates with a four‐compartment body composition model and a multislice computed tomography. Lean tissue mass, fat mass and bone density were quantified. Additionally, bone density of the hip and lumbar spine were measured. These measurements were performed before and after the intervention.

### 4.13 Stand test and supine‐to‐stand test

The stand test was used to check the relationship between cerebral oxygenation and postural stability. The test required standing for a duration of 3 min following 5 min in the supine position. During the supine‐to‐stand test for each participant, an 11‐min supine‐to‐stand test (5‐min supine followed by 6‐min quiet stance) was conducted for arterial and skeletal muscle‐pump baroreflex assessment along with postural sway control (Cattuzzo et al., 2020).

### 4.14 HDBR baroreflex test

This in‐bed protocol involved a 7‐min test session for cardiovascular function assessment and monitoring. Participants were instrumented for continuous blood pressure, heart rate and seismocardiography, which were recorded while the participant remained quietly resting in the study room. Testing was conducted at the middle and end of bedrest with data acquisition the same as the supine‐to‐stand.

### 4.15 Organization of sensory motor control test with surface and intramuscular EMG

Organization of sensory–motor control was measured using surface EMG to obtain the H reflex and the compound muscle action potential at the soleus and vastus lateralis muscles (Stålberg et al., 2019). Motor unit characteristics were measured from the M. vastus lateralis of the right leg and data analysed with DQEMG software (Jung et al., 2021)

### 4.16 Muscle protein kinetics measurements

The oral deuterium oxide (^2^H_2_O) approach was used since it is less restrictive, less invasive and a safe and effective method for measuring diurnal (day‐by‐day) rates of muscle protein synthesis (Miller et al., 2020). Ingestion of D_2_O labels exchangeable hydrogen atoms in newly synthesized non‐essential amino acids (i.e., alanine) that then incorporate in body proteins, including skeletal muscle, and negates the requirement for intravenous administration of metabolic tracers. This technique required a bolus dose of 400 mL administrated 50 mL every 1 h 30 min to avoid any dizziness experienced previously. In addition, a daily maintenance dose of 40 mL was administered to maintain the body water enrichment throughout the 26 days. The Nτ‐[^2^H_3_]‐MH infusion technique was used to measure myofibrillar protein breakdown rates (Baracos, 2015) in the postabsorptive and postprandial states. Details of the D_2_O and methyl histidine infusion techniques will be described in a future paper including the results.

### 4.17 Resting energy expenditure

Resting energy expenditure (REE) was measured by continuous indirect calorimetry using a ventilated‐hood metabolic monitor. All participants had fasted for 10 h, and allowed to rest in a supine position in a thermally neutral, quiet room for a minimum of 30 min before oxygen consumption and carbon dioxide production measurements begin. Subjects breathed under the plastic canopy for 20 min, and the average of the last 15 min was used for the calculation of the 24‐h REE based on the de Weir equation as previously described (Chevalier et al., 2011).

### 4.18 Cardiopulmonary exercise testing

Exercise testing was performed on an electronically braked cycle ergometer using a graded exercise protocol to determine peak exercise capacity by quantifying peak work, capacity of oxygen utilization (${\dot{V}}_{O_{2}}$), cardiac output and lung mechanics and function. This protocol included a warm‐up cycling of 5 min at a light workload between 0 W and approximately 50% of ${\dot{V}}_{O_{2}\mathrm{peak}}$ measured in screening tests, with the work rate varying in a pseudorandom binary sequence (Albouaini et al., 2007)

### 4.19 Cardiac and vascular ultrasound

Ultrasound imaging of the heart and main blood vessels were conducted with the Sonoscanner Orcheo Lite system (Paris, France) to provide detailed investigations of heart function, arterial wall stiffness and endothelial function.

### 4.20 Sleep quality

Sleep architecture and sleep quality were assessed by three‐electrode electroencephalogram (MUSE). Participants wore the device on the forehead for each night of measurement starting at 18.00 h and continuing until the following day at 12.00 h. We also continuously measured objective sleep quality throughout the 26‐day experimental protocol using an (Motion Watch 8 by CamNtech Ltd. Fenstanton, Cambridgeshire, UK), with data analysed using MotionWare 1.0.27 (CamnTech) to estimate sleep duration and efficiency. To measure participants’ subjective sleep quality, we used the Pittsburgh Sleep Quality Index (PSQI), a 19‐item questionnaire that assesses sleep quality (Buysse et al., 1989). Additionally, to assess participants' subjective sleep quality over the past 7 days, the PROMIS‐Sleep Disturbance questionnaire (short form) was used (Yu et al., 2012).

### 4.21 Muscle mass assessment by magnetic resonance imaging

This non‐invasive quantification of changes in muscle mass, and fat in muscle and bone marrow was performed in a whole‐body high field 3 T magnetic resonance imaging (MRI) (including magnetic resonance spectroscopy spectra) system located in the McGill University Health Centre. For the lower leg images (thigh and calf muscle) participants were measured in a supine position. For the forearm images, the right arm was scanned with the participant lying prone in a ‘superman’ position in order to avoid image artifacts. Thigh muscle cross‐sectional area and total volume were quantified (Morais et al., 2000). Despite the fact that the MRI measurement was performed on day 13 of HDT, participants maintained the supine position during the transportation to the MRI room and during the measurements. Total scanning time was about 90 min per participant, including the possible need to repeat a scan due to movement artifact.

### 4.22 Heart MRI

Cardiovascular magnetic resonance (CMR; i.e., cardiac MRI) was used to quantify myocardial volumes, mass and thus the monitoring of both overt and subclinical changes in mass, volume, function (strain) and myocardial tissue characterization (T1/T2 mapping, oxygenation‐sensitive). CMR‐derived systolic and diastolic myocardial strain parameters were assessed (Friedrich, 2017). Scanning was performed in the same instrument as above at the same time points. Total scanning time was about 40 min per participant, including possible need to repeat a scan due to movement artifact.

### 4.23 Peripheral quantitative computed tomography

Standard peripheral quantitative computed tomography (pQCT; XCT3000; Stratec Medizintechnik, Salzburg, Austria) was used to assess muscle area and muscle and marrow densities of the dominant forearm and lower leg. Participants were instructed to place their forearm and leg into the gantry and to hold still (Anderson et al., 2021). Total scanning time was about 60 min per participant, including the possible need to repeat a scan due to movement artifact.

### 4.24 High resolution pQCT

High resolution pQCT was used to measure bone micro‐architecture and volumetric density at the dominant distal radius (forearm) and tibia (leg). The participant was instructed to place their hand or leg into the machine's gantry and to hold still. The total scanning time was about 40 min per participant, including the possible need to repeat a scan due to movement artifact (Whittier et al., 2020)

### 4.25 Structural and functional neuroimaging

Brain tissue integrity (neuronal, metabolic, connectivity and cerebrovascular) was measured using a 3.0 T MRI scanner with a head coil and advanced machine‐learning techniques for image analysis (Badhwar et al., 2020). This measurement was performed at the Douglas Mental Health University Institute. For this measurement, the Canadian Dementia Imaging Protocol (www.cdip‐pcid.ca) was installed on the scanner. The 40 min protocol included 3D T1‐weighted (T1w), PD/T2w, T2*, FLAIR, DTI sequencing, to which a susceptibility‐weighted image and an arterial spin labelling sequence were added. In a second ‘functional’ session, we performed the Chronic Inflammatory Demyelinating Polyneurpathy (CDIP) resting‐state functional MRI BOLD sequence, as well as a cognitive map functional MRI task. The goal of this task was to test spatial construction and orientation, which is compromised in spaceflights and should be also affected during bedrest. The task was administered to astronauts as part of the Wayfinding Project at the Canadian Space Agency.

### 4.26 Blood volume measurement

Blood volume was measured by asking participants to breathe from a closed‐circuit containing a known quantity of carbon monoxide (approximately 40–50 mL to achieve carboxyhaemoglobin <8%; smokers have values of 5–8%) (Durussel et al., 2013). The dilution of the gas in the blood, and therefore the quantity of blood, is determined from the carboxyhaemoglobin concentration.

### 4.27 Blood and urine sampling

A standard venipuncture was performed on different days during the study (Table 4) from the antecubital vein in the arm into appropriate tubes. Thereafter blood was centrifuged, and blood sera and plasma were kept frozen in liquid nitrogen and stored at −80°C until analysed or transported to the MUHC laboratory for analysis. The blood serum and/or plasma was used for haematology and hormone analysis. In addition, circulating markers of inflammation, cardiometabolic risk factors, neurotrophic factors, markers of neural injury, muscle protein and bone turnover, and nutrition were measured.

**TABLE 4:** Volume of blood drawn during the study.

	Over 26 days				
Screening (±30 days before admission)	Baseline period (over 5 days)	Bedrest period (over 14 days)	Recovery period (over 7 days)	4‐week follow‐up	4‐month follow‐up
32 ml	109 ml	269 ml	110 ml	55 ml	49 ml

The highest blood volume collected during the 26‐day in‐hospital stay amounted to 488 mL. This amount is equivalent to a blood donation that averages 470 mL. One needs to consider that it was drawn over an extended period of time that posed no threat to these healthy subjects who have undergone a very stringent screening process. It is known that this amount of blood volume can be replaced in 24–48 h and the red blood cells in 10–12 weeks. By the time participants went to their follow‐up visits there was no risk. The total amount of blood drawn throughout the 6 months of study was 624 mL.

### 4.28 Oral glucose tolerance test (OGTT)

Participants were asked to drink 75 g of dextrose given in the form of a non‐carbonated orange‐flavoured drink after 10 h of an overnight fast and a consistent meal and fluid consumption on the previous evening (Goulet et al., 2009). The research nurse inserted an intravenous catheter into an antecubital vein of the participants before the drink. Two blood samples were taken at baseline (before the glucose drink) and at 15, 30, 60, 90, 120 and 180 min after the glucose drink. At each sample time, 2 mL of blood was taken for glucose and insulin measurements. The total blood volume collected for this test was 16 mL. We will apply the Matsuda and other indices to calculate insulin sensitivity index with higher values signifying greater sensitivity.

### 4.29 Saliva sampling

Salivary samples were used to assess free cortisol levels (Salimetrics, Carlsbad, CA, USA). Salivary samples were collected into salivettes (Sarstedt Inc. Numbrecht, Germany) 5×/day (at awakening, +30 min, +6 h, +12 h, bedtime) for 2 consecutive days. Additionally, salivary samples were collected to measure body water D_2_O enrichment, DNA methylation and oral microbiome. Samples were stored at room temperature and some at −80°C before being transported to Dalhousie University, the University of British Columbia and Waterloo University.

### 4.30 Faeces sampling

To minimize the need for invasive procedures, we exclusively used faecal samples as proxies for the gut microbiome of the study participants. Faecal samples were collected from bed pans as soon as possible after the bowel movement, then frozen and stored at −80°C before being transported to the Integrated Microbiome Resource (IMR) at Dalhousie University. There, samples were stored at −80°C.

### 4.31 Muscle needle biopsy

Biopsies were taken from the lateral portion of the M. vastus lateralis. This procedure was performed by one of the two physicians involved in the study, each having extensive expertise in performing these biopsies in frail, older and healthy individuals. The procedure, which has been used in numerous previous investigations of the hosting principal investigator, is routine in many metabolic studies and uses the percutaneous needle technique with aspiration (Chevalier et al., 2011). A portion of fresh biopsy tissue was immediately used for mitochondrial function analysis, a second portion of the biopsy was prepared for histology/immunohistochemistry, and the remaining muscle tissue was snap‐frozen in liquid nitrogen and stored at −80°C for protein and RNA analysis.

## 5 DATA ANALYSIS

For common measures across all investigators, repeated measures ANOVA was used with within‐group effects that defined the time‐course of events and between‐group effects that defined the effects of exercise as a countermeasure, provided the assumptions of normality and homoscedasticity held. Time‐by‐group interactions indicated differential changes over time between groups. When data were not normally distributed, the Kruskal–Wallis test was used or the variable was log‐transformed. Importantly, individual teams adopted different statistical approaches when analysing their specific variables. When ANOVA revealed significant differences, post‐hoc tests were conducted to identify distinct time points. Pearson's (or Spearman's, if unequal variance) correlation coefficient and multiple regression analyses related parameters. Depending on the parameter, partial correlations were performed to account for confounders.

## 6 DISCUSSION AND CLINICAL RELEVANCE

This project was a premiere in Canada as the first head down bedrest study to comprehensively, prospectively and longitudinally investigate the effects of a 14‐day bedrest and its recovery up to 4 months in the target older population. Of the 23 participants, 22 completed the study successfully. One participant dropped out on day 3 of HDBR, for adaptability and comfort reasons (difficulty staying in head‐down position, inability to pass bowel movement and requiring assistance for basic needs). During the recovery phase, two male participants from the control group encountered a heart‐related incident, specifically an A‐fib episode, on the third day. Nevertheless, the ethics committee concluded that these events were unrelated to the study and did not categorize them as adverse events. Due to these occurrences on the third day of recovery, data for these participants were absent for the 4‐week and 4‐month follow‐ups, as they were subsequently withdrawn from the study on day 3 of recovery.

The objective of this paper is to provide detailed descriptions of all tests and inpatient arrangements necessary for the study's implementation, aiming to guide other teams interested in conducting similar bedrest studies. We employed a randomized, non‐blinded design due to the intervention's requirement for an exercise countermeasure. The execution of the study involved convening meetings with all principal investigators from various teams to establish a schedule outlining the tests for each individual protocol. The study investigated the effects of bedrest on organic systems, functional capacities, physiological changes and multifaceted psychosocial factors. In a subsequent phase, anticipated collaboration among the research teams would allow for the correlation of detailed organ or physiological functions assessed by specialized experts, thereby expanding the study's scope. This approach offered both economies of scale and enhanced reliability since measurements were taken from the same individuals over time under consistent conditions.

The project addressed the influence of characteristics of an exercise programme, such as duration, training frequency and intensity, on the anticipated changes resulting from bedrest. This project proved crucial in bolstering evidence in this field through hierarchical forms of knowledge creation. The timing was opportune, as studies on bedrest in older adults had recently garnered significant attention (Di Girolamo et al., 2021), partly due to their serving as an effective model for accelerated ageing (Mulavara et al., 2018) in an era of increased life expectancy. Additionally, the focus was on longer‐duration space flights, and there was a trend toward including older astronauts compared to earlier times. Enhanced evidence was urgently necessary to bridge this knowledge gap and inform the decision‐making processes of space agencies and healthcare facilities. Furthermore, there was a need to develop, implement and evaluate activity‐based and tailored physical activity programmes for astronauts and older adults.

The evidence, once further investigated, could: (1) help astronauts during their missions and following their return to be in better health, (2) better prepare future space flights for longer and prolonged missions, (3) assist in understanding the phenomenon of deconditioning associated with hospitalizations in older adults, and (4) suggest different type of exercise intervention during prolonged hospital stay. Of even greater clinical relevance is the expectation that our exercise protocol will reduce impairments (i.e., organic systems) and optimize aptitudes, which are expected to positively influence physiological and psychosocial outcomes in older adults and potentially those undertaking prolonged spaceflight missions. While bedrest does not perfectly replicate the conditions experienced by astronauts in space, there is a notable resemblance between the effects of prolonged bedrest and the challenges faced by astronauts. Employing a bedrest model becomes essential for research purposes, allowing for a larger participant pool and enhancing statistical significance through the examination of diverse protocols. Moreover, conducting invasive procedures like biopsies and extensive blood draws poses significant challenges in a space environment. Therefore, bedrest models serve as valuable tools for gaining insights into intracellular changes that occur during periods of microgravity.

In the long term, although not measured in the proposed study, we can hypothesize that indirect societal benefits may become tangible via reduced health care costs and caregiver burden. Such benefits would be envisioned, for example, by optimizing global health in older inpatients with prolonged periods of bedrest resulting in shorter periods of hospitalization. Last, as exercise interventions have started to be implemented in hospital facilities, including them while patients are in the lying down position could maximize the benefit of exercise during the standing recovery period. Still, further evidence is needed to support the expected beneficial effects of exercise during bedrest in the long term. Therefore, this project is relevant, as it will generate the first evidence of the anticipated cardiorespiratory, musculoskeletal, endocrine–metabolic and psychological health adaptations upon completion of an exercise programme during bedrest.

Due to the wide range of the proposed evaluations, it will become possible for the first time to estimate the extent to which these adaptations translate into beneficial effects on physical and psychological health, performance and improved quality of life of both astronauts and older adults. Considering that we are planning for longer space missions and the rise of life expectancy in the world's population, these potential beneficial effects are further warranted. Furthermore, results will be relevant for rehabilitation programme administrators and policy makers since the evidence generated may justify publicly funded clinical and technological infrastructures, the creation of structured programmes incorporating exercises in nursing homes and introducing outcome measures into rehabilitation or adapted exercises in hospital centres.

## 7 TRIAL STATUS AND PUBLICATIONS

This clinical study was registered at ClinicalTrials.gov. The registration number is NCT04964999 and this is protocol version 7. Recruitment of participants started mid‐April 2021 and was completed at the end of September 2021. All participants completed the study in April 2022 including the 4 months’ follow up.

The MUHC research team oversaw the coordination, the steering committee, data management team and other individuals or groups overseeing the trial. Until today, seven paper have been published from this trial including data of the Canadian Space Agency standard measures and other parameters on bone vascularization, cognitive function, baroreflex, cardiovascular capacity and so forth (Balbim et al., 2024; Blaber et al., 2023; Hajj‐Boutros et al., 2023; Hedge, Mastrandrea et al., 2023, Hedge, Vico et al., 2023; Mastrandrea et al., 2023; Sadeghian et al., 2022).

## AUTHOR CONTRIBUTIONS

Guy Hajj‐Boutros, Vita Sonjak, Andréa Faust, Sharmila Balram, Jean‐Christophe Lagacé, Philippe St‐Martin, Donya Naz Divsalar, Farshid Sadeghian, Andrew P. Blaber, Isabelle J. Dionne, Teresa Liu‐Ambrose, Simon Duchesne, Saija Kontulainen, Olga Theou, and José A. Morais conceived the study. All authors developed the study design and contributed to the refinement of the study protocol. Guy Hajj‐Boutros, Vita Sonjak, Andréa Faust, Sharmila Balram, Jean‐Christophe Lagacé, Philippe St‐Martin, Donya Naz Divsalar, Farshid Sadeghian, and José A. Morais collected the data. All authors have read and approved the final version of this manuscript and agree to be accountable for all aspects of the work in ensuring that questions related to the accuracy or integrity of any part of the work are appropriately investigated and resolved. All persons designated as authors qualify for authorship, and all those who qualify for authorship are listed.

## CONFLICT OF INTEREST

The authors declare no conflicts of interest.
